# Long-Term Behavioral Effects of Post-weaning Social Isolation in Males and Females

**DOI:** 10.3389/fnbeh.2019.00066

**Published:** 2019-04-11

**Authors:** Deena M. Walker, Ashley M. Cunningham, Jill K. Gregory, Eric J. Nestler

**Affiliations:** ^1^Department of Neuroscience and Friedman Brain Institute, Icahn School of Medicine at Mount Sinai, New York, NY, United States; ^2^Academic IT: Instructional Technology Group, Icahn School of Medicine at Mount Sinai, New York, NY, United States

**Keywords:** adolescence, isolation rearing, anxiety, depression, reward, addiction, serotonin, dopamine

## Abstract

Adolescence is a developmental period associated with vast neural and behavioral changes which are accompanied by altered sensitivity to stimuli, both stressful and rewarding. Perturbations, especially stressful stimuli, during this period have been shown to alter behavior in adulthood. Social isolation rearing is one such perturbation. This review highlights the long-term behavioral consequences of adolescent social isolation rearing in rodents with a specific focus on anxiety- and addiction-related behaviors. Sex-specific effects are discussed where data are available. We then consider changes in monoaminergic neurotransmission as one possible mechanism for the behavioral effects described. This research on both normative and perturbed adolescent development is crucial to understanding and treating the increased vulnerability to psychiatric disorders seen in humans during this life stage.

## Introduction

As individuals transition from childhood to adulthood, they gain sexual maturity as well as the cognitive, emotional, and social skills needed to establish independence from their parents (Spear, 2000). This transition, termed adolescence, is a time of increased exploration, including increased sensation seeking, risk taking, and drug use (Steinberg, 2004; Lipari and Jean-Francois, 2013). This dynamic transition is a developmental period associated with the emergence of psychiatric disorders such as anxiety, depression, eating disorders, and schizophrenia (Kessler et al., 2005). Though it is unclear why such disorders emerge during adolescence, one hypothesis suggests a developmental mismatch between striatal driven sensation seeking (risk taking) and prefrontal inhibition of impulsivity (behavioral control). This mismatch seems to lead to a greater sensitivity to both rewarding and stressful stimuli (Casey and Jones, 2010). It has been posited that the increased sensitivity to stress during adolescence may contribute to the increased incidence of psychiatric illness at this stage in life (Grant, 1997; Grant and Dawson, 1998; Turner and Lloyd, 2004). In adults, it is clear that individual differences in stress sensitivity influence susceptibility (vs. resilience) to numerous psychiatric disorders including depression, anxiety, and substance use disorders (SUDs). Additionally, the effects of stress are exacerbated when they occur during critical developmental periods including during gestation and neonatal development (Bagot et al., 2014; Chen and Baram, 2016). Recent evidence suggests that adolescence is another key developmental window during which stressful experiences may result in long-term, even permanent alterations to brain structure and function (Burke et al., 2017). Hence, research into the neurobiological underpinnings of adolescence may elucidate a basic understanding of normative social, emotional, reproductive, and cognitive development as well as contribute to the understanding of health risks and disorders that impact this life stage.

## Adolescence and the Development of Social Reward

**Figure 1:**
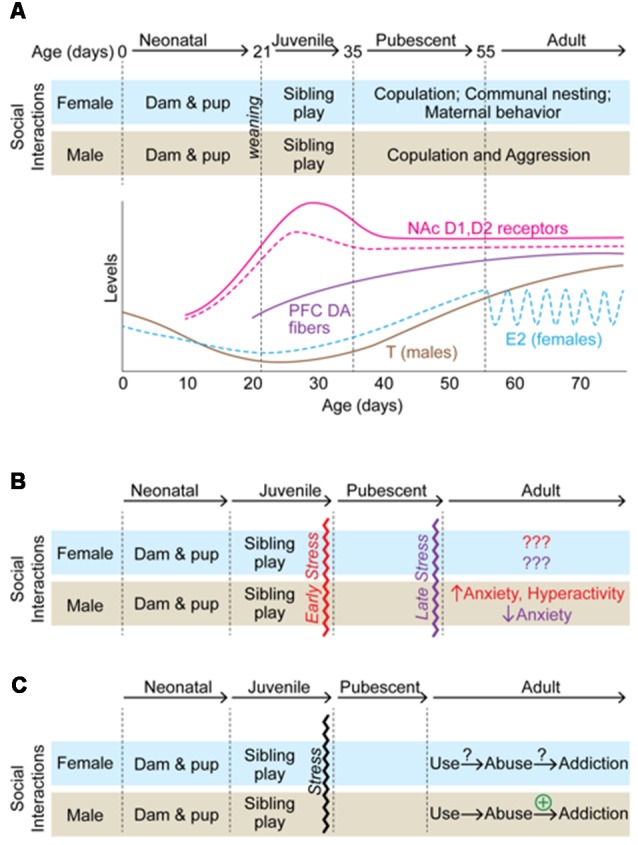
A summary of how adolescent isolation rearing alters behavioral outcomes in male and female rodents. **(A)** A schematic of developmental markers from birth to adulthood in male (solid lines) and female (dashed lines) rodents. Social interactions/behaviors are indicated at each development milestone and correspond to changes in gonadal hormones and changes within the dopamine (DA) system. **(B)** Effects of isolation rearing on anxiety-related behaviors. In males, stress early in adolescence has opposite effects on anxiety-related behaviors than late adolescent isolation. **(C)** Effects of isolation rearing on addiction-related behaviors. While little information is available regarding how adolescent isolation rearing affects females, it increases addiction-related behaviors in males.

Adolescence is the developmental process that surrounds pubertal onset and the attainment of reproductive capacity. While puberty and adolescence are often used synonymously, they are very different processes. Puberty is defined as the attainment of adult reproductive competency and involves the activation of the hypothalamic-pituitary-gonadal axis. Adolescence, on the other hand, is the period surrounding pubertal onset and includes a longer window in which an organism prepares for independence from their parents. Specific behaviors emerge during this time which facilitate this transition including play behavior, altered social interactions, and increased exploration (Figure 1A; Steinberg, 2004; Lipari and Jean-Francois, 2013). During this period, there is a qualitative shift in the salience of social reward in particular (Spear, 2000). In humans, a hallmark of adolescence is a change in the quantity and quality of social interactions with peers and family which includes an increase in time spent with peers (Larson et al., 1996). Rather than turning to their family, adolescents rely on their contemporaries for social support and become increasingly sensitive to treatment by their peers (Ladd et al., 2014). Evidence suggests that social interactions during adolescence can influence the development and maintenance of maladaptive behaviors in adults (Patterson et al., 1992; Hankin et al., 1998). In fact, negative peer influences are a strong predictor of depression in adolescence and adolescent depression is associated with a life-time risk for major depressive disorder (MDD; Thapar et al., 2012). It is easy to think of the re-organization of a social structure during adolescence only in the context of the emergence of various psychiatric disorders (Kessler et al., 2005). However, it is important to note that the restructuring of social organization during adolescence is necessary for social species to develop behavioral strategies that are essential for survival in adulthood (Gopnik et al., 2017). Additionally, the association of such reorganization with pubertal onset is thought to decrease the likelihood of inbreeding within a social group by increasing exposure to genetically distinct individuals at a time when reproductive behaviors emerge (Lawson Handley and Perrin, 2007). If perturbations disrupt this crucial developmental window, long-term consequences in behavior can persist into adulthood and influence an organism’s social and sexual competency (Schulz et al., 2009; McCormick and Green, 2013; McCormick et al., 2013a,b).

Similar to humans, adolescent changes in social interactions are prevalent in rodents. Many of these changes are sex-specific. Adolescent male rats exhibit a greater preference for social stimuli in a conditioned place preference (CPP) test when compared to adults (Douglas et al., 2004; Yates et al., 2013) or to females (Douglas et al., 2004; Weiss et al., 2015). Male rodents also place a greater value on peer-directed activities including play behaviors (Pellis and Pellis, 2017). However, it is important to note that this effect is most pronounced in males deprived of social interactions. Additionally, a peer-paired chamber negates cocaine (Zernig et al., 2013) and amphetamine (Yates et al., 2013) induced CPP in adolescent males but not females (amphetamine only; Weiss et al., 2015). These data suggest that there are striking sex differences in responses to social reward in adolescent rodents with males displaying a greater sensitivity to social reward than females. It is thought that adolescent-specific social experiences result in permanent neural and hormonal changes essential for the development of cognitive processes, which enable effective coping in adulthood (Spear, 2000). Therefore, the observed sex differences in sensitivity to social reward may profoundly influence neural circuitry involved in reward as well as sex differences in reward-associated behavior in adulthood.

## Evidence of Increased Stress Sensitivity During Adolescence

Mounting evidence suggests that adolescent animals are more sensitive to both acute and chronic/repeated stressors, and this enhanced sensitivity may contribute to an increased vulnerability to psychiatric disorders that arise during the adolescent period. The convergence of multiple factors results in a unique state with regard to stress sensitivity. First, adolescent rodents exhibit prolonged stress-induced corticosterone responses when compared to adults (Romeo, 2010) or pre-weanlings (Sapolsky and Meaney, 1986). Numerous studies have identified a protracted adrenocorticotropic hormone (ACTH) and corticosterone response in adolescent (~postnatal day P30) male and female rodents, which is induced with both physical and psychological stressors (Romeo, 2013). Similar effects have been observed in humans. Adolescent boys and girls (15–17 years) display a greater stress-induced cortisol increase when compared to younger individuals (9–13 years). However, it should be noted that adults were not included in these studies, making it difficult to determine if this effect is specific to adolescence (Gunnar et al., 2009; Stroud et al., 2009). Not only are adolescents more sensitive to stressors, they also sensitize to repeated stressful stimuli. Unlike adults, which habituate to a repeated stressor, adolescent rodents display an enhanced corticosterone response to repeated stressors (Romeo et al., 2006; Lui et al., 2012). Therefore, under chronic stress conditions, the adolescent brain is exposed to a vastly different hormonal environment than adults. Furthermore, evidence suggests that the adolescent brain may be more sensitive to the effects of corticosterone. Specifically, corticosterone-induced gene expression of glutamate receptor subunits in the hippocampus is enhanced in late adolescence (P42) when compared to adult animals (P67; Lee et al., 2003). Finally, key stress-sensitive brain areas, including the amygdala (AMY), hippocampus (HIP), and prefrontal cortex (PFC; Giedd and Rapoport, 2010), and associated neurotransmitter systems continue to develop throughout adolescence (Figure 1A; Walker et al., 2017). These brain regions express glucocorticoid receptors at relatively high levels throughout adolescence (Vazquez et al., 1998; Romeo, 2013; Dziedzic et al., 2014). Taken together, these data suggest that the adolescent brain is exceptionally sensitive to stressful experiences and, given the crucial developmental changes that occur during adolescence, stress exposure could result in long-term alterations in the brain and behavior.

## Adolescence and Social Stress

As mentioned above, social reward develops during adolescence. Additionally, the adolescent organism may be more sensitive to stress. Therefore, it follows that adolescence is a period of enhanced sensitivity to social stress, specifically, disruptions to normal development of social structure could influence behaviors in the long-term. Indeed, there is ample evidence that disruption of social structures during adolescence influence reward-, anxiety-, and depression-associated behaviors in rodent models. For example, social instability stress (e.g., a stressor in which animals are housed with different cage mates over a period of time, thus preventing the establishment of stable social hierarchies during adolescent development) has been shown to influence depressive- and anxiety-like behaviors as well as reward-associated behaviors including drug preference in adulthood (McCormick and Green, 2013). In addition to social instability stress, a number of stressors that have been utilized to study the long-term effects of social disruptions on adult behavior (Burke et al., 2017). One of the most prominent social stressors utilized is post-weaning social isolation stress in which an animal is housed individually throughout adolescent development. Although protocols differ, in this paradigm an animal is placed in a cage by itself throughout the adolescent period so that it is deprived of social interactions during this developmental window. In most paradigms, the timing of isolation coincides with the initiation and enhancement of play behavior (Figure 1A). Play behavior, or play fighting, occurs in many species. It is a form of non-serious fighting in which animals engage in pinning, rolling, and attacking the nape of the necks of their cage-mates. Partners take turns attacking and defending against the behaviors and rarely do the bouts turn into serious aggressive attacks. In fact, many of the behaviors involved in play resemble pre-copulatory behaviors and some hypothesize that play behavior is important for preparing animals for adult social behaviors, including defense and copulation, by providing an opportunity to practice such behaviors as juveniles (Pellis and Pellis, 2017). Play behavior is specific to the juvenile/adolescent period. In rats, play behavior emerges around P16 and increases until it peaks on ~P30, after which it declines into adulthood (Baenninger, 1967; Panksepp, 1981). The fact that play behavior is specific to the adolescent period and resembles those behaviors necessary for reproductive success suggests it may be crucial for the proper development of social reward. Thus, stressors that prevent exposure to play, such as adolescent social isolation, may result in long-term behavioral deficits in social reward and behaviors associated with reward in general.

Post-weaning social isolation stress has been used widely to understand how social stress influences behaviors in the adolescent model with conflicting results. The differences are more than likely due to differences in experimental design, length of separation, and the timing of behavioral testing (Lukkes et al., 2009b). This review highlights the long-term behavioral consequences of social stress during the adolescent period with a specific focus on social isolation stress. For the long-term effects of other forms of social stress please see Burke et al. (2017).

The social isolation model has the potential to become all the more relevant with the recent changes in social structure that have emerged due to the use of smartphones and social media. Recent evidence in humans suggests that adolescent males and females who spend more time on social media are lonelier than their peer counterparts (Primack et al., 2017). This changing dynamic within the human population makes understanding the consequences of disrupted adolescent social structures critical, now more than ever. While it has been used extensively in the field to understand how early-life experience might influence adult behavior, there currently is no standard for the implementation of adolescent social isolation stress. Generally, this protocol is considered to be relevant to loneliness, which has been shown to be a risk factor for anxiety, depression, and addiction in numerous studies. Most isolation paradigms include a period of isolation beginning around P21–P30 and ending after 3–8 weeks. Animals are either shipped to the facility on ~P21 or born onsite. At weaning, the animal is housed alone in a standard cage with access to food and water. Generally, sensory cues are not limited, in that they can see, hear, and smell other animals in the colony and experience normal husbandry procedures. However, some studies have utilized opaque cages to limit stimulation still further. Given the vast neuronal, endocrine, and behavioral changes that are associated with adolescence, the timing of isolation is critical for the long-term behavioral effects. For example, given the progression of play behavior mentioned above (see “Evidence of Increased Stress Sensitivity During Adolescence” section), starting isolation on P21 vs. P28 can lead to critical differences in social reward development. Additionally, there are known strain and sex differences in the timing of puberty (Nelson et al., 1990; Krewson et al., 2004; Parent et al., 2005; Zhou et al., 2012; Corre et al., 2016), suggesting that these small but significant differences in isolation paradigms may also coincide with vastly different hormonal and neuroendocrine profiles across studies. Therefore, it is critical to develop an understanding of normative adolescent development in order to fully understand the consequences of adolescent experience on adult behaviors.

Additionally, in most cases, animals are not resocialized before behavioral testing (Lukkes et al., 2009b; Burke et al., 2017). This adds a level of uncertainty to the analysis, as it is difficult to determine if the effects are due to social isolation throughout their lives or if the adolescent period is indeed a sensitive period to the effects of adolescent stress. Throughout the review, the term “isolation rearing” will be used when discussing studies that do not resocialize and those studies that resocialize are noted to help clarify effects that may be sensitive to adolescent-specific perturbations. In an attempt to address this issue, two well-known studies sought to determine a “critical window” for social isolation stress on anxiety- and reward-associated behaviors. Einon and Morgan (Einon and Morgan, 1977) first addressed this issue by comparing different isolation windows (P16–P25; P25–P45; and P90–P180) to group-housed (GH) controls on anxiety-related behaviors and neophobia. Isolated individuals took longer to enter the center in an open field test regardless of the timing of isolation, an effect that was reversed by resocialization, suggesting that isolation stress influences open field exploration independent of the developmental window. However, it should be noted that the effect was most pronounced when the isolation took place beginning on P25, suggesting an interaction between stress and adolescent period. Also, animals isolated from P25 to P45 fail to habituate to the presence of a novel object even after resocialization. Together, these data suggest that isolation during adolescence has pronounced effects on novelty seeking which cannot be reversed by resocialization (Einon and Morgan, 1977). Regarding reward-associated behavior, Whitaker et al. (2013) showed that male rats isolated from P21 to P43, but not P21–P28 or P42–P63, showed a greater preference for EtOH and amphetamine (AMPH), altered neuronal activity in the ventral tegmental area (VTA), greater sensitivity of VTA neurons to corticotropin releasing factor (CRF), and altered glutamatergic signaling in the VTA. They went on to demonstrate that resocialization from P43 to P63 did not reverse the effects on VTA neuronal activity. However, behavior was not examined after resocialization (Whitaker et al., 2013). These data provide valuable information and could help standardize the adolescent social isolation protocol in the field and determine sensitive windows of susceptibility for social stress in rodents.

## Social Isolation Stress Disrupts Adult Behavior

### Anxiety-Related Behaviors

The adolescent period is a crucial time for corticolimbic development. Alterations in dendritic pruning have been observed in almost all regions of the mesocorticolimbic circuitry, and connectivity between these critical brain regions is maturing during the adolescent process (Figure 1A; Walker et al., 2017). While further research is necessary, there is evidence that stress during adolescence disrupts connectivity between key mesocorticolimbic regions and alters dendritic pruning (Eiland and Romeo, 2013). Experimental disruption of such structure/connectivity, either through lesion studies (Winter et al., 2007; Koenigs and Grafman, 2009) or other types of circuity manipulations (Felix-Ortiz et al., 2013, 2016; Felix-Ortiz and Tye, 2014; Bagot et al., 2015) in adults alters anxiety- and depression-related behaviors in rodents. Therefore, it is not surprising that studies characterizing the behavioral effects of adolescent stress have focused on anxiety-related behaviors. Generally, exposure to physical and psychological stress in adolescence results in increased anxiety-related behavior (Romeo, 2017) and social stress, in particular, has a profound effect on adult behavior (Burke et al., 2017). However, studies investigating the effects of social isolation stress have produced inconsistent results (Table 1). These differences are likely due to differences in experimental design. For example, Holson et al. (1991) showed that the anxiogenic effects of isolation rearing could be eliminated by handling the isolated animals a few times per week. Others have suggested that differences in light cycle or time of testing may contribute to the inconsistent effects (Arakawa, 2018). However, even given these inconsistencies some themes are beginning to emerge from the data (Table 1) and many of the results highlight striking sex differences (Figure 1B) in the effects of social isolation rearing in particular.

**Table 1:** Behavioral effects of isolation rearing/adolescent social isolation on anxiety-related behaviors.

Behavioral test	Period of isolation	Resocialized?	Age of testing	Species	Sex	Behavioral effect in isolated (compared to GH controls)	Consequence of SI	References
Elevated Plus Maze	P21–P42	No	P42	Rats (NA)	M	↓ exploration	Anxiogenic	Parker (1986)
	P21–~P80		~P80	Rat (LH)		↓ time in open arm		Wright et al. (1991)
	P21–P85		~P85	Rat (LE)				Pritchard et al. (2013)
	P21–P112		~P112	Rat (SD)				Weiss et al. (2004)
	P28–P72		~P77	Rat (LE)				McCool and Chappell (2009), Chappell et al. (2013), Yorgason et al. (2013), Karkhanis et al. (2014) and Skelly et al. (2015)
	P21–P77		~P77	Rat (SD)		NE	NE	Zhao et al. (2009)
	P45–~P130		~P130	Rat (W)		↑ time in open arms	Anxiolytic	Thorsell et al. (2006)
	P21–P51	Yes	~P80	Rat (LH)	M	↓ time in open arm	Anxiogenic	Wright et al. (1991)
	P30–P50*		P80	Rat (SD)		↑ time in open arm; after restraint stress	Anxiolytic	Weintraub et al. (2010)
	P21–P112	No	~P112	Rat (SD)	F	NE	NE	Weiss et al. (2004)
	P28–P50		P51–P53	Rat (SD)				Jahng et al. (2012)
	P31–~P70		~P70	Rat (LE)				Butler et al. (2014)
	P30–P50*	Yes	P80	Rat (SD)	F	NE	NE	Weintraub et al. (2010)
Open Field (locomotor)	P19–P105	No	P105	Rat (W)	M	↑ locomotor activity	Hyperactive	Gentsch et al. (1981)
	P21–P77		~P77	Rat (SD)				Zhao et al. (2009)
	P21–P105		P105	Rat (W)				Heidbreder et al. (2000)
	P22–~P58		~P58	Rat (LH)				Dalrymple-Alford and Benton (1981)
	P28–~P200		~P200	Rat (W)				Archer (1969)
	P28–P72		~P77	Rat (LE)				Skelly et al. (2015)
	P28–P72		~P77	Rat (LE)		↑ distance traveled		Skelly et al. (2015)
	P21–P90		~P90	Rat (LE)		NE Locomotor	NE	Gardner et al. (1975)
	P45–~P130		~P130	Rat (W)				Thorsell et al. (2006)
	P21–P112		~P112	Rat (SD)		NE distance traveld	NE	Weiss et al. (2004)
	P21–P100		~P100	Rat (SD)		↓ locomotor activity	Hypoactive	Holson et al. (1991)
	P21–P120		P120	Rat (LE)				Holson et al. (1988)
	P21–P42	Yes	P56	Rat (SD)	M	↓ locomotor activity	Hypoactive	Lukkes et al. (2009c)
	P21–P51		~P80	Rat (LH)		↑ locomotor activity	Hyperactive	Wright et al. (1991)
	P22–~P58	No	~P58	Rat (LH)	F	↑ locomotor activity	Hyperactive	Dalrymple-Alford and Benton (1981)
	P28–P50		P51–P53	Rat (SD)				Jahng et al. (2012)
	P28–P50		P51–P53	Rat (SD)		↑ distance traveled		Jahng et al. (2012)
	P28–P42		P42	Rat (W)		NE	NE	Archer (1969)
	P31–~P70		~P70	Rat (LE)		NE		Butler et al. (2014)
	P21–P112		~P112	Rat (SD)		NE distance traveld	NE	Weiss et al. (2004)
	P21–P100		~P100	Rat (SD)		↓ locomotor activity	Hypoactive	Holson et al. (1991)
	P28–~P200		~P200	Rat (W)				Archer (1969)
Open Field (Center Point)	P21–P120	No	P120	Rat (LE)	M	NE latency to enter center	NE	Holson et al. (1988)
	P22–~P58		~P58	Rat (LH)				Dalrymple-Alford and Benton (1981)
	P21–P42	Yes	P56	Rat (SD)	M	↓ entries into center; brightly lit	Axiogenic	Lukkes et al. (2009c)
	P45–~P130	No	~P130	Rat (W)	M	↑ time in center	Anyolytic	Thorsell et al. (2006)
	P16–P45		P45	Rat (LH)	M and F combined	↑ latency enter center	Anxiogenic	Einon and Morgan (1977)
	P25–P90		P90					
	P16–P25	Yes	P45	Rat (LH)	M and F combined	NE latency enter center	NE	Einon and Morgan (1977)
	P25–P45		P90					
	P22–~P58	No	~P58	Rat (LH)	F	NE latency to center	NE	Dalrymple-Alford and Benton (1981)
Novel Object Exploration	P16–P45	No	P45	Rat (LH)	M and F combined	NE novel object investigation	NE	Einon and Morgan (1977)
	P25–P90		P90			fail to habitulate to novel object	Anxiogenic	
	P16–P25	Yes	P45	Rat (LH)	M and F combined	NE on novel object investigation	NE	Einon and Morgan (1977)
	P25–P45		P90			fail to habitulate to novel object	Anxiogenic	
	P31–~P70	No	~P70	Rat (LE)	F	NE	NE	Butler et al. (2014)

NA, not available; SD, Sprague-Dawley; LE, Long Evans; LH, Lister Hooded; W, Wistar; NE, no effect; M, male; F, female; NSF, novelty suppressed feeding. *In this study animals were exposed to additiona restraint stress after SI; 1X on P50 and from P73–P80.

#### Elevated Plus Maze

**Figure 2:**
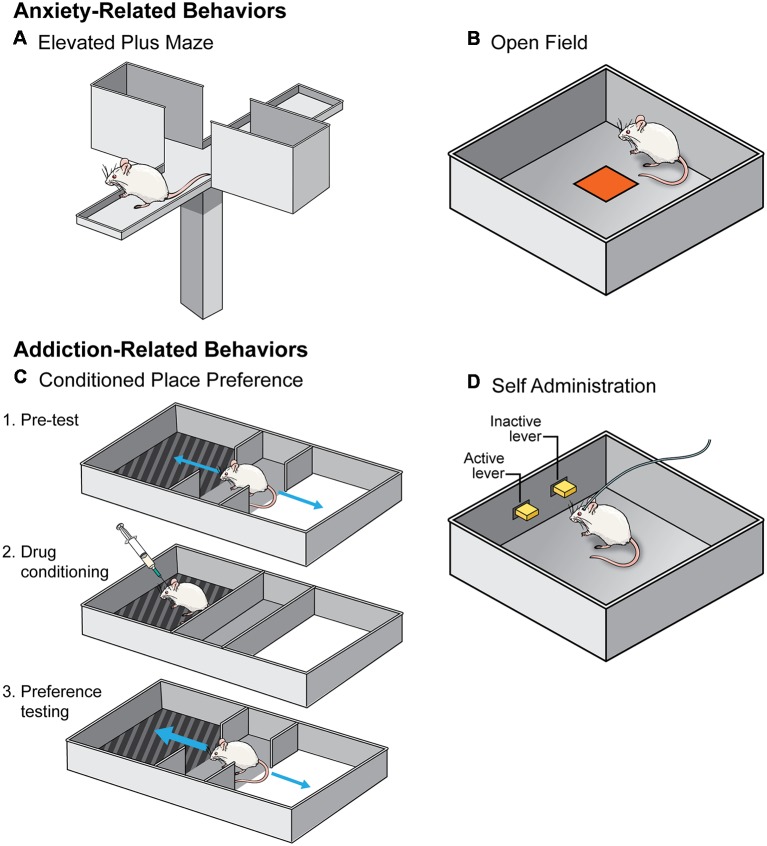
A schematic of the behavioral tests utilized to assess the effects of social isolation rearing on anxiety- (top) and addiction- (bottom) related behaviors. **(A,B)** Anxiety-related behavioral tests include elevated plus maze **(A)** and open field **(B)**. An animal is considered less anxious if they spend more time in the open arm of an elevated plus maze and the center of an open field. **(C,D)** Addiction-related behavioral tests include conditioned place preference (CPP; **C**) and self-administration (SA; **D**). An animal who forms a preference for the side of a chamber where they received a drug in CPP is considered to find that drug more rewarding **(C)**. In SA paradigms **(D)**, animals learn that pressing the active lever but not the inactive lever results in an injection of a drug of abuse. Animals are thought to find drugs of abuse more rewarding if they learn the operant task more quickly.

In general, studies investigating how social isolation rearing influences behavior on an elevated plus maze have produced consistent results (Table 1). During testing, an animal is placed on an “X” shaped maze, that stands ~3 feet off the floor, and is composed of two enclosed arms and two open arms, all of which the animal can explore freely. It is assumed that an animal who spends more time exploring the open arms is less “anxious” because this region of the maze is exposed and is, therefore, more dangerous (Figure 2A; Lezak et al., 2017). However, this interpretation should be viewed with caution, particularly when investigating sex differences in behavior. For example, females spend more time in the open arms than males, however, careful analysis of this behavior indicated that this is due to increased activity and not decreased anxiety- or fear-related behavior (Fernandes et al., 1999).

Isolation rearing beginning in early adolescence (P21–P28) consistently decreases time spent in the open arm of an elevated plus maze in male rats (Parker, 1986; Wright et al., 1991; Weiss et al., 2004; McCool and Chappell, 2009; Chappell et al., 2013; Pritchard et al., 2013; Yorgason et al., 2013; Karkhanis et al., 2014; Skelly et al., 2015), an effect that cannot be reversed with resocialization (Wright et al., 1991). However, the timing of the stress may be critical, as males isolated on or after P30 display less anxiety-like behavior, as indicated by more time spent in the open arms of the maze (Thorsell et al., 2006; Weintraub et al., 2010). Interestingly, this coincides with the decline of play behavior in group-housed rats (Baenninger, 1967; Panksepp, 1981), and suggest that the anxiogenic effects of early adolescent isolation rearing may be due to the lack of play interactions in male rats. In females, however, isolation rearing has no effect on behavior in an elevated plus maze (Weiss et al., 2004; Weintraub et al., 2010; Jahng et al., 2012; Butler et al., 2014) regardless of whether the animals were resocialized following stress (Weintraub et al., 2010), suggesting that the effects of adolescent rearing and adolescent social isolation are more potent in male rodents.

#### Open Field Behavior

As with elevated plus maze, investigation into how isolation rearing affects open field behavior are relatively consistent (Table 1). During testing, an animal is allowed to explore a novel environment, usually a large plastic box. Animals in a novel environment often display an increase in locomotor behavior, interpreted as an effect of the stress of being in a new context, but typically habituate during the test. Animals thought to be more anxious tend not to habituate as quickly and will, therefore, display an increased exploration measured *via* increased distance within the open field and an increase in locomotor behavior. Determining the latency, or how long it takes an animal, to enter into the center of the arena is another measurement of anxiety-like behavior in an open field test. Generally, open spaces are unsafe for rodents, therefore, an animal is considered less anxious if they enter the center more readily (Figure 2B; Lezak et al., 2017). Again, caution should be used when interpreting results from this test, especially in the context of sex differences in behavior. Studies have found that females are more hyperactive and hence enter the center of an open field more readily than males. Therefore, the traditional interpretation of open field testing may not be an accurate measure of anxiety, especially in females.

The effects of isolation rearing on locomotor activity are inconsistent in both males and females (Table 1). Although the majority of studies have found that male rats reared in isolation are hyperactive in an open field test (Archer, 1969; Dalrymple-Alford and Benton, 1981; Gentsch et al., 1981; Heidbreder et al., 2000; Zhao et al., 2009; Skelly et al., 2015), an appreciable number found that isolation rearing has no effect on locomotor activity (Gardner et al., 1975; Weiss et al., 2004; Thorsell et al., 2006) and some have found that isolation rearing leads to *hypoactivity* (Holson et al., 1988, 1991). Similarly, resocialization has produced conflicting results with Wright et al. (1991) showing an increase and Lukkes et al. (2009c) showing a decrease in locomotor behavior in males. In females, the inconsistencies are even more apparent. However, this is likely due to the fact that there are far fewer studies in females. The limited number of studies in female rats has shown that isolation rearing increases (Dalrymple-Alford and Benton, 1981; Jahng et al., 2012), decreases (Archer, 1969; Holson et al., 1991) or does not affect (Archer, 1969; Butler et al., 2014) locomotor behavior. It is unclear why social isolation has produced such inconsistent results, but differences in experimental design including length of isolation, lighting conditions during testing, and the open field apparatus used likely to contribute. As with other behavioral indicators of anxiety-like behavior, there appears to be a window in which isolation must occur for any effect to be observed. Archer (1969) found that isolation for 2 weeks (P28–P42) was not enough to affect anxiety-related behaviors in females (males not studied). However, after 24 weeks of isolation, a significant decrease in locomotor behavior was observed in females, whereas an increase in locomotor behavior was observed in males (Archer, 1969). This is consistent with multiple studies indicating that isolation must occur for at least 3 weeks for any behavioral effects to be observed (Whitaker et al., 2013; Burke et al., 2017).

As mentioned above, another measure of anxiety-like responses in an open field is the time it takes for an animal to enter the center of the apparatus and total time spent in the center during testing (Figure 2B). Once again, the effects of isolation rearing on this behavioral measure are inconsistent in males and females. In males, isolation in early adolescence (beginning on P21) does not affect the latency to enter the center of the apparatus (Dalrymple-Alford and Benton, 1981; Holson et al., 1988), but does decrease the number of times an animal will enter the center after resocialization (Lukkes et al., 2009a). However, isolation late in adolescence (P45–P130) results in male rats spending more time in the center of the field (Thorsell et al., 2006). Interestingly, these results mirror those observed in the elevated plus maze data discussed above and suggest that isolation in early adolescence is anxiogenic, while isolation later in adolescence is anxiolytic in male rats. To our knowledge, only one study has investigated this behavioral measure in females and found no effect on the latency to enter the center of the open field (Dalrymple-Alford and Benton, 1981), further highlighting the need for research into the effects of isolation rearing on females.

#### Other Anxiety/Fear-Related Behaviors

While it is beyond the scope of this review to cover all anxiety-related behaviors affected by adolescent isolation rearing, it should be noted that similar effects have been observed in fear conditioning, social interaction testing, pre-pulse inhibition, and startle response (For reviews see McCormick and Green, 2013; Burke et al., 2017). Of interest to this review are the studies which have found similar sex-specific effects of social isolation rearing on fear conditioning in rats. In this behavioral paradigm, animals are trained to associate a tone with a fear-inducing stimulus (usually a foot shock). Freezing in response to a stimulus is a behavioral marker of fear in rodents. Therefore, once an animal undergoes training, freezing behavior is observed after an animal is exposed to the paired tone within the conditioned context (McAllister, 1971). Social isolation from P21 to P42 resulted in males freezing longer in response to the conditioned tone on P77 (Lukkes et al., 2009a). Additionally, males isolated from P28 to P77 took longer to extinguish their response to the conditioned tone when compared to group-housed counterparts (Skelly et al., 2015). However, isolation reared females (P21–P112) froze less than their control counterparts when re-exposed to the conditioned context (Weiss et al., 2004). These results highlight once again that there are important sex differences in adolescent development that are disrupted by isolation rearing in sex-specific ways. Further research is necessary to not only characterize normative adolescent development of anxiety-related behaviors in males and females but also to understand how each sex might be affected by stress at key developmental time points.

#### Summary of Anxiety-Related Behaviors

Taken together, these data suggest that social isolation rearing has profound effects on anxiety-related behaviors, as best as they can be inferred, in male rodents and subtle, if any, effects in females (Figure 1B). Importantly, these effects are not alleviated by resocialization, suggesting that restricting social stimuli at a time when social reward is heightened results in a permanent change in exploratory and novelty-seeking behaviors. While there are inconsistencies in many of the behavioral endpoints, these differences are likely due to differences in experimental design and highlight the need for standardized stress protocols for investigating the long-term effects of adolescent social isolation on anxiety-related behaviors. Even with these inconsistencies, it seems clear that the timing of stress is important. Social isolation during early adolescence is anxiogenic in male rats, whereas isolation later in adolescence is anxiolytic. Further studies are necessary to determine the mechanisms underlying these effects and if these effects hold up across species. For example, do the anxiogenic effects hold up in species that engage in less adolescent play? Similarly, much more work is necessary to understand how adolescent social isolation affects female behavior. As it stands, the effects of social isolation rearing are more subtle in females than in males. However, given that many of the behavioral tests for anxiety were developed in males and do not seem to be indicative of anxiety-like behavior in females, it may be that new tests need to be developed for testing the effects of adolescent social isolation in females.

### Addiction-Related Behaviors

SUDs—namely, drug addiction—are defined as the continued use of drugs of abuse despite negative consequences (American Psychiatric Association, 2013). It is a complex disorder characterized by cycles of behavior. In humans, a SUD begins with accelerated use after initial exposure to a drug of abuse. As the SUD becomes more severe, a person may enter a cycle of addiction that includes uncontrolled drug use followed by periods of attempted abstinence (withdrawal) and then all too often by a return to drug use (relapse; Koob and Simon, 2009). It is well established that early life adversity influences susceptibility to addiction in adulthood (Pena et al., 2014). While the initial mechanisms of action of drugs of abuse are numerous, they all converge on the mesolimbic dopamine (DA) circuitry to increase the release of DA in the nucleus accumbens (NAc) from a population of neurons within the VTA. As mentioned above, evidence suggests that this VTA—NAc circuit, as well as VTA innervation of other forebrain limbic regions, is developing during the adolescent period (Figure 1A; Walker et al., 2017) and that exposure to stressful stimuli can disrupt connectivity between these brain regions. Thus, adolescent stress may interfere with the normative development of the mesocorticolimbic system and impact the effects of drugs of abuse within the brain’s reward circuitry.

#### Conditioned Place Preference Testing

There is convincing evidence that isolation rearing influences an animal’s ability to form a preference for a drug-paired chamber in the CPP paradigm (Table 2). In CPP testing, an animal is trained to associate a context with a specific drug of abuse by exposing them to the drug or a vehicle control within different chambers of an CPP apparatus. These chambers differ in sensory modalities (e.g., color or pattern on the walls, types of floors, intensity of lighting, etc.). After training sessions, an animal is allowed access to both chambers of the apparatus and the time spent on the drug-paired side is determined. An animal who spends more time in the drug-paired chamber is said to have formed a preference for the drug. This preference is thought to be indicative of the reinforcing properties of a drug (Figure 2C; Belin, 2012).

**Table 2:** Behavioral Effects of isolation rearing/adolescent social isolation on addiction-related behaviors conditioned place preference.

Drug	Isolation	Rehoused?	Age of testing	Species	Sex	Dose	Isolated	GH control	Stage of addiction	References
Amphetamine	P21–P51	No	~P51	Rat (LE)	M	0.031 mg/kg	NE (no preference)	No Preference		Schenk et al. (1986)
						0.0625 mg/kg				
						0.125 mg/kg				
						0.25 mg/k				
						0.5 mg/kg				
	P21–P42		P42	Rat (SD)		5 mg/kg	↑ preference compared to GH	No Preference		Whitaker et al. (2013)
							longer to extinguish AMPH CPP (Day 14)	Extinguished on Day 9		
							↑ acquisition of CPP compared to GH (Day 1)	Acquired on Day 3		
	P42–P63		P63	Rat (SD)		5 mg/kg	NE (no preference)	No Preference		Whitaker et al. (2013)
Cocaine	P21–P51	No	~P51	Rat (LE)	M	0.31 mg/kg	↓ preference compared to GH	Preference		Schenk et al. (1986)
						0.625 mg/kg	NE (no preference)	No Preference		
						1.25 mg/kg				
						2.5 mg/kg				
	P23–P43		P47	Rat (SD)		3 mg/kg	NE (no preference)	No Preference		Zakharova et al. (2009)
						5 mg/kg	NE (Preference)	Preference		
						10 mg/kg	↑ preference compared to GH	No Preference		
EtOH	P21–P28	No	P28	Rat (SD)	M	0.5 g/kg	NE (no preference)	No Preference		Whitaker et al. (2013)
	P21–P42		P42	Rat (SD)		0.5 g/kg	↑ preference compared to GH	No Preference		Whitaker et al. (2013)
Morphine	P21–P49	No	P49	Rat (LH)	M	1 mg/kg	↓ preference compared to GH	Preference		Wongwitdecha and Marsden (1996)
	P21–P63		P63			1.5 mg/kg				Wongwitdecha and Marsden (1995)
	P21–P49		P49			5 mg/kg				Wongwitdecha and Marsden (1996)
	P21–P63		P63							Wongwitdecha and Marsden (1995)
**Self-Administration**									
Amphetamine	P21–P55	No	P55 Food training; P61 SA	Rat (SD)	M and F	0.03 mg/kg/inf	↑ lever pressing	no acquisition of drug SA; ↓ lever pressing after switched from food to AMPH	intake	Bardo et al. (2001)
						0.1 mg/kg/inf	NE; lever pressing maintained when switched from food to AMPH	lever pressing maintained after switched from food to AMPH		
						0.03 and 0.1 mg/kg/inf	NE on PR testing	NE	motivation	
Cocaine	P21–P80	No	~P80	Rat (LH)	M	0.083 mg/kg/inf	↑ acquisition	No acquisition	acquisition	Howes et al. (2000)
	P21–P63		~P63	Rat N/A		0.1 mg/kg/inf	↑ in % animals that acquired	No acquisition		Schenk et al. (1987)
	P21–P55		~P55	Rat (SD)		0.1 mg/kg/inf	NE on acquisition	Acquisition		Gipson et al. (2011)
	P21–~P70		~P70	Rat (LE)		0.25 mg/kg/inf	↑ in % animals that acquired	No acquisition		Smith et al. (2014)
	P21–P80		~P80	Rat (LH)		0.25 mg/kg/inf	NE on acquisition	Acquisition		Howes et al. (2000)
	P21–P63		~P63	Rat (N/A)		0.5 mg/kg/inf	↑ in % animals that acquired	No acquisition		Schenk et al. (1987)
	P21–P55		~P55	Rat (SD)		0.5 mg/kg/inf	↑ acquisition	No acquisition		Gipson et al. (2011)*
	P21–~P70		~P70	Rat (LE)		0.75 mg/kg/inf	↑ acquisition; ↑ in % animals that acquired	No acquisition		Smith et al. (2014)
	P21–P63		~P63	Rat N/A		1 mg/kg/inf	↑ in % animals that acquired	No acquisition		Schenk et al. (1987)
	P21–P150		~P150	Rat (LH)		1.5 mg/kg/inf	↓ acquisition compared to GH	Acquisition		Phillips et al. (1994)
	P21–P80		~P80	Rat (LH)		1.5 mg/kg/inf	↓ acquisition	Acquisition		Howes et al. (2000)
	P21–~P70		~P70	Rat (LE)		1.5 mg/kg/inf	↑ in % animals that acquired	~40% acquired		Smith et al. (2014)
	P21–P43	Yes	~P90	Rat (LH)		0.083 mg/kg/inf	↑ acquisition	No acquisition		Baarendse et al. (2014)
	P21–P43	Yes	~P90	Rat (LH)		0.25 mg/kg/inf	NE	acquisition		Baarendse et al. (2014)
	P21–P150	No	~P150	Rat (LH)	M	0.023–1.5 mg/kg/inf	shifted dose response curve to right compared to GH	acquisition	dose respone	Phillips et al. (1994)
	P21–P43	Yes	~P90	Rat (LH)		dose response: 0.03–0.5	NE			Baarendse et al. (2014)
	P21–P63	No	P63	Rat (LE)	M	0.04 mg/kg/inf	↑ lever pressing	intake = saline	intake	Boyle et al. (1991)
			P63			0.08 mg/kg/inf	NE	intake > saline		Boyle et al. (1991)
			~P63			0.1 mg/kg/inf	NE	intake = saline		Schenk et al. (1987)
			P63			0.16 mg/kg/inf	NE	intake > saline		Boyle et al. (1991)
			P63			0.32 mg/kg/inf	NE	intake > saline		Boyle et al. (1991)
			~P63			0.5 mg/kg/inf	↑ lever pressing	intake = saline		Schenk et al. (1987)
			P63			0.64 mg/kg/inf	NE	intake > saline		Boyle et al. (1991)
			~P63			1 mg/kg/inf	↑ infusions over GH	intake = saline		Schenk et al. (1987)
	P22–P55		~P55 Food training; P61 SA	Rat (SD)		0.5 mg/kg/inf	↑ active lever presses	N/A		Ding et al. (2005)
			~P55 Food training; P61 SA			0.5 mg/kg/inf	↑ infusions compared to GH	N/A		Ding et al. (2005)
	P21–P43	Yes	~P90	Rat (LH)		0.083 mg/inf	↑ infusions over GH	N/A		Baarendse et al. (2014)
	P21–P55	No	~P55	Rat (SD)	M	0.1 mg/kg/inf	↑ escalation	no escalation	escalation	Gipson et al. (2011)
						0.5 mg/kg/inf	no escalation but start at higher (ceiling effect?)	escalate intake from day 8 to 21 (Different control group EE)		Gipson et al. (2011)*
	P21–P43	Yes	~P90	Rat (LH)	M	PR at 0.083 and 0.25 mg/inf	higher breakpoint compared to GH		motivation	Baarendse et al. (2014)
Morphine	P21–P61	No	~P66	Rat (SD)		0.8 mg/ml	advanced acquisition* (oral route of exposure)		acquisition	Marks-Kaufman and Lewis (1984)
							consumed more during relapse (oral route of exposure)		intake	

N/A, not available; EtOH, ethanol; SD, Sprague-Dawley; LE, Long Evans; LH, Lister Hooded; W, Wistar; NE, no effect; inf, infusion; PR, progressive ratio; SA, self-administration; AMPH, amphetamine. *Different control group–compared to environmentally enriched group; did not include a socially enriched group for this experiment.

Studies investigating how isolation rearing influences CPP to drugs of abuse indicate that isolation rearing increases sensitivity to psychostimulants (Table 2), an effect that appears to be dose-dependent. This phenomenon is not observed for opiates, in fact, a reduction in sensitivity in response to isolation rearing has been reported. Specifically, isolation rearing increases the preference for amphetamine (Whitaker et al., 2013) and cocaine (Zakharova et al., 2009) when compared to the group housed controls at higher drug doses (5 and 10 mg/kg). However, this effect was not observed at lower doses of cocaine (0.31–2.5 mg/kg) or amphetamines (0.031–0.5 mg/kg; Schenk et al., 1986), or when isolation rearing commenced on P42 (Whitaker et al., 2013). On the other hand, while socially reared animals formed a strong preference for morphine at low (1 and 1.5 mg/kg) and high (5 mg/kg) doses, isolated animals did not (Wongwitdecha and Marsden, 1995, 1996), suggesting an interaction between social context during development and pharmacology. It should be noted that the number of studies is quite small and further research is necessary to determine if this finding is indeed specific to psychostimulant vs. opiate drugs of abuse. Additionally, there is a dearth of information regarding how isolation rearing or adolescent social stress in general influences drug reward in females (Figure 1C; Table 2).

#### Self-Administration

Investigations into the effects of social isolation rearing on drug reward have mainly focused on self-administration (SA; Table 2). In this behavioral test, animals are given the opportunity to administer drugs of abuse under their own volition. Often, animals are implanted with a jugular vein catheter and connected to a pump used to deliver drugs directly into the bloodstream after the animal has pressed a lever or nose-poked. Through operant conditioning, animals learn to discriminate between an “active” lever (drug infusion) and “inactive” lever (no consequence) with the drug serving to reinforce the lever-pressing behavior (Figure 2D; Belin, 2012). Generally speaking, isolation rearing increases the acquisition of SA (Marks-Kaufman and Lewis, 1984; Schenk et al., 1987; Phillips et al., 1994; Howes et al., 2000; Green et al., 2002; Baarendse et al., 2014), and increases intake of numerous drugs of abuse (Marks-Kaufman and Lewis, 1984; Schenk et al., 1987; Boyle et al., 1991; Bardo et al., 2001; Ding et al., 2005), with the difference most apparent at low doses (for specifics see Table 2). Importantly, this effect is not reversed by resocialization from P42 to P90, at least with regard to cocaine SA (Baarendse et al., 2014).

Cocaine SA, in particular, has been extensively used to study the effects of social isolation rearing on different aspects of drug-taking behaviors. Acquisition of SA is defined as the transition to a regular pattern of intake as indicated by an animal reliably pressing the drug-paired over the inactive lever. It is interpreted as a behavioral marker of a drug’s reinforcing properties. From a translational perspective, the rate of acquisition could provide insight into the progression of SUDs in humans. Variables that increase the rate of acquisition are considered to be risk factors for SUDs, while those that delay acquisition are considered to be protective against SUDs in humans (Belin, 2012). To our knowledge, studies on the effects of isolation rearing on cocaine SA behaviors in females have not been investigated (Table 2). However, studies in males reveal that isolation rearing increases the rate of acquisition of cocaine SA at low doses in males [between 0.083 and 1 mg/kg/infusion (inf)] with relative consistency. It is important to highlight that, in each of these studies, the group-housed control animals did not acquire, suggesting that low doses of cocaine are more reinforcing after isolation rearing (Schenk et al., 1987; Howes et al., 2000; Gipson et al., 2011; Smith et al., 2014). A more complicated picture emerges when the dose of cocaine is increased to the point where control animals also acquire cocaine SA. In these cases, if the group-housed control animals acquire SA, the rate of acquisition is delayed in isolated animals (Phillips et al., 1994; Howes et al., 2000) or not affected by housing condition (Howes et al., 2000; Gipson et al., 2011). It is tempting to interpret these data as an increase in sensitivity to cocaine in isolated animals. However, this is not supported by the few dose-response experiments that have been conducted. Phillips et al. (1994) showed that isolation rearing results in a rightward shift in the dose response to cocaine (0.023–1.5 mg/kg/inf), suggesting that isolated males were *less* sensitive to the reinforcing effects of cocaine. In support of this, a recent study (Baarendse et al., 2014), which included a period of resocialization from P42 to ~P90, showed that isolation specifically during the adolescent period had no effect on the dose-response curve in adult males (0.03–0.5 mg/kg/inf). These inconsistencies are likely due to differences in experimental design and reflect the high variability of complex behaviors in control populations. Additionally, these results highlight the need for standardized protocols for SA.

As with acquisition, lever pressing and intake are often interpreted as a behavioral marker of the reinforcing properties of drugs of abuse. Generally speaking, isolation rearing in males increases the intake of cocaine (Schenk et al., 1987; Boyle et al., 1991; Ding et al., 2005), amphetamine (Bardo et al., 2001), and morphine (Marks-Kaufman and Lewis, 1984), again suggesting that isolated animals may display differences in the sensitivity to drugs of abuse. Most of these effects have been observed in cocaine SA where similar to acquisition, the effects are dose dependent and must be interpreted in relation to group-housed controls in each experiment. At low doses (0.08–0.32 mg/kg/inj), isolated animals show no differences in intake when compared to group-housed control animals (Schenk et al., 1987; Boyle et al., 1991). At higher doses (0.5 and 1 mg/kg/inj) isolated animals take more cocaine when compared to control animals (Schenk et al., 1987; Boyle et al., 1991; Ding et al., 2005; Baarendse et al., 2014). Similar to acquisition, these results must be interpreted in the context of the group-housed control behaviors. When the control animals show a preference for cocaine over saline (Schenk et al., 1987; Boyle et al., 1991; Ding et al., 2005; Baarendse et al., 2014), there is no difference in intake between isolated and group-housed animals. On the other hand, when there is no difference in intake between saline and cocaine in the control group, isolated animals tend to lever press more than their control counterparts (Schenk et al., 1987; Boyle et al., 1991; Ding et al., 2005; Baarendse et al., 2014), suggesting that there are substantial differences in reward sensitivity across the groups. Importantly, to our knowledge, no study has found that group-housed controls consume more than isolated animals for any drug of abuse (Table 2).

Upon first glance, it might seem that isolation rearing increases sensitivity to the reinforcing properties of drugs of abuse. However, these results should be interpreted with caution. First, isolation rearing has been reported to increase not only active but *inactive* lever pressing (Boyle et al., 1991; Ding et al., 2005; Baarendse et al., 2014) and isolated animals will lever press more for saline (Schenk et al., 1987). Therefore, increases in lever pressing/intake may be driven by isolation rearing effects on activity levels, especially at low drug doses (see “Anxiety-Related Behaviors” section; Table 1). This could explain the dose-response effects observed in acquisition as well. At low doses, hyperactive animals may be exposed to drug as a function of more interactions with the levers, whereas at higher doses when a drug serves as a stronger reinforcer both group housed and isolated animals acquire cocaine SA. In support of this, there is a body of evidence that suggests that isolated animals are more sensitive to the locomotor effects of psychostimulants (reviewed in Fone and Porkess, 2008). Thus, a feedforward effect would be predicted, as increased locomotor response would increase interactions with the levers and increase exposure to drug. Regardless of the mechanism driving this finding, it is noteworthy that attention deficit disorder with hyperactivity is a risk factor for SUDs (Wilens, 2004). Thus, isolation rearing may provide a translational model for understanding the behavioral, neural, and cellular/molecular mechanisms underlying susceptibility to SUDs.

While understudied, other behavioral measures may provide more information regarding how isolation rearing influences the progression of addiction-like behaviors in animal models. Escalation is considered a model of the transition from recreational to compulsive drug taking (Belin, 2012). Under short access conditions (~2 h sessions), an animal controls its intake to maintain a stable blood level throughout the session. However, when moved to long-access (~6 h) conditions, animals escalate their intake, especially in the first hour, and no longer defend their preferred plasma drug concentrations (Ahmed and Koob, 1998). Gipson et al. (2011) investigated escalation at two different doses in male rats isolated from P21 to P55 and found that isolated, but not group housed, animals escalated their intake of cocaine at 0.1 mg/kg/inf after being switched to long-access. However, at a higher dose (0.5 mg/kg/inf), group-housed males escalated their intake, an effect not observed in the isolated animals. In this experiment, isolated animals received more injections on the first day of long-access testing. This level of intake was similar to the control animals on the final day of testing, suggesting that isolated animals may have reached a “ceiling” in the number of responses and that isolation results in a loss of control in drug consumption (Gipson et al., 2011). It should be highlighted that this is only one study and further investigation is necessary to determine how social stress in adolescent males and females influences escalation of consumption of numerous drugs of abuse.

Finally, motivation to gain access to a drug can be assessed by changing the criteria for reward during an SA session. In a progressive ratio scheduling paradigm, increasing response ratios are used to determine when an animal will no longer “work” for the reinforcer, termed the “break-point.” A higher break-point is interpreted as an increased motivation for reward (Roberts et al., 1989; Richardson and Roberts, 1996). To our knowledge, only one study has investigated how adolescent isolation affects motivation for cocaine reward in this paradigm. Baarendse et al. (2014) found that males isolated for 3 weeks (P21–P42) followed by several weeks of resocialization had a higher breakpoint under a progressive ratio schedule for cocaine at both low (0.083 mg/inf) and high (0.25 mg/inf) doses when compared to their group housed control counterparts.

#### Summary of Addiction-Related Behaviors

Taken together, these data suggest that isolation rearing affects susceptibility to drug addiction through two mechanisms: (1) both CPP and SA data suggest that reward sensitivity is altered by isolation rearing; and (2) hyperactivity induced by isolation rearing may interact with a sensitized reward circuity to induce susceptibility to addiction later in life. Further research is necessary to determine if similar pharmacological effects are observed in SA paradigms as those observed in CPP paradigms between psychostimulants vs.opiates (and other classes of drugs of abuse) and if social isolation rearing in females results in similar behavioral effects to those observed in males.

## Social Isolation and Disruption of Monoamine Systems

Monoamines are a class of neurotransmitters that are derived from aromatic amino acids. Generally speaking, these molecules modulate the excitatory and inhibitory effects of glutamate and GABA, respectively. Monoamines including serotonin (5-hydroxytryptamine or 5-HT), DA, norepinephrine, and epinephrine, are important signaling molecules that influence higher cognitive function, reward integration, and arousal (Lipton, 1974). While all monoamines play a role in anxiety and addiction, 5-HT and DA have been implicated as crucial to stress and reward processing. Specifically, increased 5-HT function in the brain underlies therapeutic responses to most classes of antidepressant medications. While it is hypothesized that susceptibility to anxiety and depression in humans may relate to differences in 5-HT function (Blier, 2016), considerable evidence shows no such relationship (Nestler et al., 2002). On the other hand, DA has been extensively implicated in reward processing and it is hypothesized that dysregulation of the DA system is an important component of SUDs (Nutt et al., 2015). Accumulating evidence suggests that these two monoaminergic systems are crucial for the environmental influence on behavior in adolescence (Burke et al., 2017) and both are important for CNS development (Sodhi and Sanders-Bush, 2004). Given their roles in regulating emotion and stress responses, and their importance in CNS development, it is not surprising that many studies have focused on the effects of isolation rearing on 5-HT and DA signaling dynamics.

### Social Isolation and Dysregulation of Serotonin Signaling

5-HT neurons are concentrated in the midbrain raphe nuclei and such neurons in the dorsal raphe project throughout the brain, including mesocorticolimbic regions essential for reward processing (Sodhi and Sanders-Bush, 2004). There is an interplay between 5-HT and DA signaling which appears to influence anxiety- and reward-related behaviors (Blier, 2016). In rodents, the vast majority of serotonergic development occurs from embryonic day 14–17, suggesting that the 5-HT system is in place prior to adolescent development. However, one study found that isolation rearing increased 5-HT fiber density in the dorsal striatum and amygdala in gerbils (Lehmann et al., 2003), suggesting that 5-HT development might continue into adolescence or that there are important species differences in the developmental trajectory of the 5-HT system. Additionally, 5-HT exerts growth factor-like effects and has been shown to influence the development of other neurotransmitter systems (Sodhi and Sanders-Bush, 2004). This evidence suggests that 5-HT signaling during adolescence may be crucial for the proper development of the adolescent brain and adult behavior. Indeed, recent evidence suggests that altering serotonergic signaling, specifically during adolescence, increases anxiety-like behavior in adulthood (Ansorge et al., 2007; Donaldson et al., 2014). The importance of 5-HT in brain development coupled with its influence on anxiety-related behaviors suggest it as a key regulator of the long-term effects of isolation rearing.

**Figure 3:**
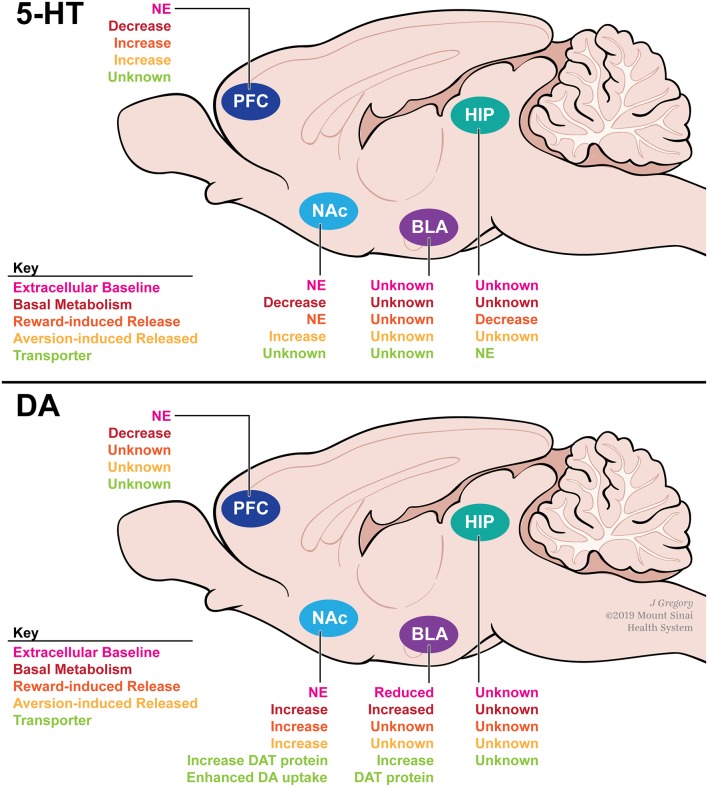
A summary of how adolescent social isolation rearing alters serotonin (top) and DA (bottom) dynamics within the reward circuitry of rodents. Social isolation rearing has been shown to alter metabolism (red) and release (orange and yellow) of serotonin in the PFC, NAc and HIP in a region-specific manner. Similarly, social isolation rearing has been shown to alter baseline DA (pink) as well as metabolism (red), release (orange and yellow) and reuptake through actions on DAT (green) in a region-specific manner. Abbreviations: PFC, prefrontal cortex; NAc, nucleus accumbens; BLA, basolateral amygdala; HIP, hippocampus; NE, no effect; DAT, dopamine transporter.

Studies investigating the effects of isolation rearing on 5-HT signaling have found brain region-specific effects on basal 5-HT release and turnover (Figure 3A). In the NAc, a region important for the integration of reward signaling, isolation rearing has no effect on basal extracellular 5-HT levels (Howes et al., 2000), but decreases basal turnover of 5-HT (Heidbreder et al., 2000), suggesting that isolation rearing alters 5-HT dynamics within the NAc. Additionally, exposure to an aversive stimulus (foot shock; Fulford and Marsden, 1998a), but not a rewarding stimulus (cocaine; Howes et al., 2000), increases the release of 5-HT into the NAc after isolation rearing. In the PFC, a critical region of executive function and decision making, a similar effect to NAc is observed on basal 5-HT; isolation rearing has no effect on basal extracellular 5-HT levels but decreases its metabolism, as indicated by decreased 5-HIAA levels (Holson et al., 1991). This finding suggests once again that 5-HT dynamics, including reuptake, in this brain region may be affected by social isolation.

Region-specific differences emerge when investigating stimuli-induced 5-HT release (Figure 3A). In the PFC, aversive (footshock; Lapiz et al., 2003) and rewarding (amphetamine and para-chloroamphetamine; Dalley et al., 2002) stimuli attenuate 5-HT release compared to group-housed controls. In the hippocampus, little is known regarding how isolation rearing affects basal 5-HT levels. However, para-chloroamphetamine-induced 5-HT release is decreased after isolation rearing (Muchimapura et al., 2002). Further investigation within the hippocampus revealed that 5-HT reuptake was not affected by isolation rearing, suggesting that 5-HT transporter (SERT) function is unaffected (Muchimapura et al., 2002). These data suggest that isolation rearing alters both basal and stimulus-induced 5-HT signaling in a region-specific manner. Importantly, these region-specific differences may be due, in part, to differences in receptor sensitivity across the brain. Numerous studies have shown that total binding and affinity of multiple 5-HT receptor subtypes are altered by isolation rearing (reviewed in Fone and Porkess, 2008). The reported effects of isolation rearing on the serotonergic system have important implications for the long-term behavioral consequences of adolescent stress. Given the importance of 5-HT in controlling anxiety- and depression-related behaviors, these data provide a possible mechanism for the effects of isolation rearing on anxiety discussed above.

### Social Isolation and Dysregulation of Dopamine Signaling

As stated earlier, the mesolimbic DA system is characterized by a collection of DA-releasing neurons within the VTA that project to the dorsal striatum, NAc, PFC, HIP, and basolateral amygdala (BLA), among other regions. There is substantial evidence that VTA DA neuronal projections undergo vast developmental changes during adolescence, which can be disrupted by exposure to drugs of abuse and stress in rodents (Walker et al., 2017). Specifically, in male mice, DA axonal projections grow through the NAc to establish connections within the PFC. Disruption of this process results in excessive axonal growth to the PFC and decreased connectivity between the VTA and NAc (Reynolds et al., 2018). Normative development of this system is crucial for proper function for reward learning. Thus, disruption of these connections by stress during adolescence can have long-term behavioral consequences regarding motivation and reward. This work highlights the sensitivity of DA circuitry to environmental stimuli during adolescence. However, to our knowledge, no study has investigated how isolation rearing affects DA neuronal connectivity in adults. Instead, most studies have focused on how isolation rearing alters DA release into important reward-associated regions in adulthood.

Similar to the 5-HT system, isolation rearing alters DA release in a region-specific manner (Figure 3B). In male rats, isolation rearing has no effect on extracellular DA levels in the NAc (Jones, 1992; Fulford and Marsden, 1998b; Hall et al., 1998; Howes et al., 2000; Karkhanis et al., 2014). However, basal DA turnover is increased (Hall et al., 1998), suggesting that DA dynamics including release and uptake may be altered after isolation rearing. Additionally, electrical stimulation (Yorgason et al., 2013), footshock (Fulford and Marsden, 1998b; Lapiz et al., 2003), amphetamine (Hall et al., 1998; Lapiz et al., 2003; Yorgason et al., 2016), EtOH (Karkhanis et al., 2014), and cocaine (Yorgason et al., 2016) all increase DA release into the NAc in isolated animals when compared to group-housed controls. Importantly, DA depletion in the NAc can reverse the observed behavioral phenotypes following isolation rearing in male rats (Powell et al., 2003). These results suggest that isolation rearing modulates DA signaling within the NAc that may have an important impact on behavior. In the PFC, basal DA is unchanged by isolation rearing (Holson et al., 1991; Dalley et al., 2002; Powell et al., 2003), however, turnover is decreased (Heidbreder et al., 2000). In the BLA, a reward-associated region implicated in reward valuation, isolation rearing reduces basal DA concentrations (Karkhanis et al., 2015), which are coupled with increased DA turnover (Heidbreder et al., 2000). Interestingly, these changes in basal DA concentrations may have an effect on electrophysiological properties within the amygdala, as social isolation rearing has been shown to decrease cellular activity within the extended amygdala (Adams and Rosenkranz, 2016). Further functional studies are necessary to determine how social isolation rearing might affect cellular physiology throughout the reward circuitry. Additionally, unlike the 5-HT system, isolation rearing does not affect DA receptor sensitivity or expression. To our knowledge, no study has investigated how isolation rearing affects expression or activity of the DA receptor-1 (Drd1). However, several studies have found that isolation rearing has no effect on Drd2 mRNA, protein, or activity levels in the NAc, PFC, HIPP, or AMY of male rats (Del Arco et al., 2004; Malone et al., 2008; Yorgason et al., 2013). These findings may be species-specific, as a recent study in male mice found that Drd2 mRNA and protein levels are increased in the NAc, but not PFC or HIPP, after social isolation rearing (Li et al., 2017).

Unlike the 5-HT system, these region-specific differences may be due in part to differences in DA transporter (DAT) function. DAT is a membrane-spanning protein that pumps DA from the synaptic cleft back into the cytosol of DA neurons. DAT is the immediate protein target for all psychostimulant drugs of abuse, and its function reportedly influences clinical depression and alcoholism (Salatino-Oliveira et al., 2018). Studies have found that isolation rearing increases baseline DAT protein levels in the dorsal striatum, NAc, and BLA when compared to group-housed controls (Karkhanis et al., 2015; Yorgason et al., 2016). Additionally, DAT protein levels are induced in the NAc in response to three daily injections of cocaine in isolated males. This effect was not observed in animals reared in an enriched environment (Zakharova et al., 2009). Altered DAT protein levels may impact the rate at which DA is removed from the synapse. Studies investigating DAT activity have focused on changes within the NAc. Yorgason et al. (2013) demonstrated that DA uptake was enhanced in isolation reared male rats at baseline. In a follow-up study, this effect was further enhanced by administration of psychostimulants including, methylphenidate, a stimulant used to treat ADHD, in the NAc in isolated male rats (Yorgason et al., 2016). Overall, these data suggest that the region-specific differences seen in DA signaling may be due, in part, to DAT function. However, further research is required to understand how DAT function is altered in regions other than the NAc.

### Summary of Effects of Isolation on Monoamines

To date, studies have found that isolation rearing changes both 5HT and DA signaling. Further research is necessary to fully integrate these neurotransmitter effects with the behavioral consequences of isolation rearing and future studies must include more research on the monoaminergic effects of isolation rearing on females to develop a complete understanding the dysregulation of these systems. Finally, regulation of the brain’s monoaminergic systems must be integrated with the regulation of numerous other classes of neurotransmitters, neurotrophic factors, and cytokines that together control brain development, structure, and function.

## Summary and Conclusions

We are only beginning to understand the complex interplay of neural networks, endocrine signaling, and environmental inputs that drive normative adolescent development. These complicated interactions are a highly coordinated process that results in specific adult-typical behaviors which are often directed at increasing the likelihood of reproduction and offspring survival. Therefore, stressful events during this sensitive window of development would be predicted to have long-term behavioral consequences. This review has highlighted how disruptions in social structure during adolescence alters anxiety- and reward-associated behaviors and points to a few emerging themes throughout. First, the timing of social isolation stress results in different behavioral outcomes and suggests that disruption of play behavior during early adolescence may have profound effects on male anxiety in adulthood (Figure 1B). Second, isolation rearing in males increases susceptibility to addiction-related behaviors in males (Figure 1C) and this may be the result, in part, of interactions of the effects of isolation on anxiety and locomotor behaviors. Third, far more research is needed in females to assess how adolescent stress may influence adult behaviors. Fourth, changes in serotonergic and dopaminergic systems may contribute to the behavioral consequences of isolation rearing, although research is needed into the contributions of many other signaling mechanisms. Fifth, many studies have compared isolation rearing to different paradigms of environmental enrichment, and it would be important to understand how the latter influences adolescent development as well. Finally, while it is clear that adolescence is critical for expression of adult behaviors, the field would benefit greatly from further research into the development of the adolescent brain and circuitry as well as the mechanisms by which neural, endocrine, and environmental information is coordinated to produce a functional system. These investigations would be instrumental for better understanding of the psychiatric disorders that emerge during adolescence, thus leading to novel therapeutics and interventions in vulnerable populations.

Together, the data highlight three areas of focus for the field that would greatly enhance our understanding of the interplay between adolescent social experience and psychiatric disorders in adulthood. First, the field would benefit greatly from a clear description of normative adolescent brain development across several rodent and primate species. This would provide valuable information regarding sensitive periods when regions, circuitry and molecules might be disrupted due to adolescent isolation rearing. Most studies utilizing social isolation to date have subjected rodents to isolation throughout the adolescent period and it is apparent that the timing of isolation is crucial for the behavioral outcomes. Second, the field is greatly lacking in studies investigating how females respond to adolescent isolation rearing. Given the vast sex differences in adolescent development and stress responses in general, it is likely that there are sex-specific effects of isolation rearing and these must be incorporated into future studies. This work could also be leveraged to understand how differences in the timing of developmental milestones (e.g., puberty) might influence the long-term behavioral effects of adolescence social stress. Finally, the field is lacking in mechanistic studies indicating how adolescent experience reshapes molecular, synaptic and circuit processes throughout the brain to influence future behavioral responses. Given the importance of adolescence as a key period for the reprogramming of the reward-circuitry and enhanced vulnerability for the development of psychiatric disorders, further studies investigating the mechanism driving such programming would provide valuable insight into the cellular and molecular mechanisms underlying psychiatric disorders.

## Author Contributions

DW, AC and EN all wrote and commented on the manuscript. JG commented on the manuscript and made all of the figures.

## Conflict of Interest Statement

The authors declare that the research was conducted in the absence of any commercial or financial relationships that could be construed as a potential conflict of interest.
